# Traditional Knowledge, Use, and Management of *Moringa oleifera* Among the Mijikenda Community in Kilifi, Kenya

**DOI:** 10.3390/plants13243547

**Published:** 2024-12-19

**Authors:** Boniface Mwami, Anna Maňourová, Prasad S. Hendre, Alice Muchugi, Vladimir Verner, Patrick Kariuki, Naji Sulaiman, Zbynek Polesny

**Affiliations:** 1 Department of Crop Sciences and Agroforestry, Faculty of Tropical AgriSciences, Czech University of Life Sciences Prague, Kamýcká 129, 165 00 Praha-Suchdol, Czech Republic; mwami@ftz.czu.cz; 2 Department of Agricultural Sciences, South Eastern Kenya University, P.O. Box 170, Kitui 90200, Kenya; 3 Department of Plant Protection Biology, Swedish University of Agricultural Sciences, SE-230 53 Alnarp, Sweden; 4 Department of Forest Botany, Dendrology and Geobiocoenology, Mendel University in Brno, Zemedelska 3, 613 00 Brno, Czech Republic; 5 World Agroforestry (ICRAF), United Nations Avenue, Gigiri, P.O. Box 30677, Nairobi 00100, Kenya; 6 Department of Economics and Development, Faculty of Tropical AgriSciences, Czech University of Life Sciences Prague, Kamýcká 129, 165 00 Praha-Suchdol, Czech Republic; 7 Geothermal Energy Training and Research Institute (GeTRI), Dedan Kimathi University of Technology (DeKUT), Along Nyeri–Mweiga Road, P.O. Box 657-10100, Nyeri 10100, Kenya; 8 University of Gastronomic Sciences, Piazza Vittorio Emanuele II 9, 12042 Pollenzo, Italy

**Keywords:** East Africa, ethnomedicine, food security, phytonutrients, traditional knowledge

## Abstract

Although *Moringa oleifera* Lam. (Moringaceae) is a multipurpose tree with remarkable nutritional and therapeutic benefits, it is undervalued and neglected in Kenya, as the local people associate it with famine and poverty. The present study aims to assess and document the traditional knowledge on use and management as well as production constraints of the species among the Mijikenda community in Kilifi County, Kenya. We found that the plant is locally used as food and medicine for various ailments, including diabetes, high blood pressure, ulcers, stomach aches, and body pains. In addition, the plant is used for fencing and as a source of fuel. Watering and pruning were found to be the main management practices of the plant. Slightly more than half (51.3%) of the respondents reported pests as a constraint in growing the plant, while the bitter taste (60.5%) and small leaves (36.8%) were deterrents to its consumption and harvesting, respectively. More than half (55.3%) of the respondents had between one and five trees in their compounds, indicating a low preference for the plant compared to major crops. The plant species is undervalued compared to locally grown major crops, highlighting the need for concerted efforts to raise awareness of its potential benefits and address the production challenges.

## 1. Introduction

The world’s tropical and subtropical regions are home to the tropical tree *Moringa oleifera* Lam., which belongs to the monotypic family Moringaceae [1]. There are 13 species in this genus, and 11 of them are native to Africa, which are *M. arborea* Verdc, *M. rivae* Chiov, *M. borziana* Mattei, *M. pygmaea* Verdc., *M. longituba* Engl., *M. stenopetala* (Baker f.) Cufod., *M. ruspoliana* Engl., *M. ovalifolia* Dinter and A. Berger, *M. drouhardii* Jum., *M. hildebrandtii* Engl., and *M. peregrina* Fiori. The other two species, *M. concanensis* Nimmo and *M. oleifera*, are only found in Asia [2]. Compared with the other species, *M. oleifera* is the most known, used, and studied [3,4,5]. Even though it is indigenous to India, it has become naturalized in many African countries as it tolerates and adapts to a wide range of environmental conditions [6].

Unlike *M. oleifera*, which is endemic to the Indian subcontinent, *M*. stenopetala, *M. perigrina*, *M. drouhardii*, and *M. hildebrandtii* are indigenous to Africa and are used as food and medicine [7,8]. *Moringa oleifera*, thereafter named moringa (its local name), was introduced in Kenya by Indians at the beginning of the 20th century and is currently grown in drylands of the coastal region, Baringo, and lower eastern areas [9,10]. The plant grows to a height of up to 12 m, with pinnate leaves, flowers in panicles, zygomorphic, pentamerous, bisexual, and capsule-type fruits with three-winged oily seeds [11]. It produces pendulous pods that are about 20–60 cm long and resemble those of long beans [12]. Moringa has a tuberous taproot that penetrates deep into the soil to absorb water from deeper soil profiles, enabling the tree to thrive during moderate drought periods, contrary to many commercial crops [13]. Taxonomically, most contemporary systems, including the APG IV system [14], place the monotypic family Moringaceae, which includes only the genus Moringa, in the order Brassicales. Molecular studies have revealed a close relationship between Moringaceae and Caricaceae [14,15]. Prior to these molecular analyses, morphological classifications placed Moringaceae either in Brassicales or Sapindales due to the family’s distinctive morphological diversity [16]. The plant performs well in many types of soil but does best in sandy loams and slightly alkaline clay loam soils owing to their good drainage [17]. It is propagated by seeds or stem cuttings and requires minimal management practices such as watering, particularly for young trees, weeding, pruning, and pest control [18].

The species performs best with an annual rainfall of 500 to 1500 mm and an altitude of 0 to 1800 m above sea level [17], but low rainfall of less than 250 mm is unsuitable for the efficient cultivation of the plant [17,19]. Research indicates that under favorable growing conditions, moringa can produce a high biomass of 4.2 to 8.3 metric tons/hectare, subject to factors such as seasons, planting density, soil factors, fertilizer use, irrigation, and harvesting frequencies [20,21].

Previous studies have demonstrated a wide range of nutritional and medicinal benefits of the plant, hence the nickname “miracle tree” [22]. All parts of the plant, including the roots, leaves, seeds, bark, gum, and flowers, are used to treat ailments like hypertension, diabetes, stomach pains, and arthritis [18,23]. The plant parts exhibit pertinent medicinal properties such as antimicrobial, hypotensive, hypoglycemic, immunomodulatory, and anti-inflammatory activities [24,25]. Additionally, moringa is nutritionally valuable and has the potential to prevent and mitigate hidden hunger [26]. Many African communities use various parts of the plant, including flowers, fruits, immature pods, and seeds, as human food [23,27,28,29,30,31,32]. Moringa contains significant amounts of vital nutrients (g/100 g) necessary for healthy living; for instance, its leaves contain proteins (10.74–30.29), carbohydrates (41.2 g), fats (5.2 g), and fiber (12.5 g) [33,34]. It also possesses other crucial nutrients (mg/100 mg), such as vitamins A (6.8 mg), B1 (0.3), B2 (20.5), C (15.2), calcium (2003), potassium (1317) 368, and iron (0.85) [25,34,35]. The immature pods contain about 46.78% fiber and 19.34% protein content, while flowers possess palmitic, linoleic, and oleic acids, which have vital nutritional properties [36].

The plant produces a high-quality oil (43.56%) commercially known as “Ben oil”, which is extracted from the seeds of the tree [37]. This oil is a potential substitute for olive oil due to its superior fatty acid composition (>70% oleic acid) and has potential applications as biodiesel and a lubricant for machinery [38]. Additionally, it is widely used in cosmetics [38,39,40,41]. After oil extraction, the remaining seed cake can be utilized in wastewater treatment as a natural coagulant and organic fertilizer to enhance agricultural productivity [41,42,43]. Furthermore, the plant is used as animal feed for bovines, goats, poultry, and fish due to its nutritional benefits, rapid growth, and adaptability to various climates and soil conditions [44,45,46].

Despite its immense nutritional, medicinal, industrial, and agricultural benefits, moringa remains neglected, undervalued, and underutilized in Kenya. Its use is significantly lower than other locally grown crops, largely due to a lack of awareness about its potential nutritional benefits [47]. Additionally, moringa is often associated with famine, hence its nickname “famine food”, contributing to its low production and consumption [48]. However, moringa cultivation and utilization could serve as a potent alternative to address food insecurity in Kilifi, a region with high levels of malnutrition and hunger [49]. Previous studies have primarily focused on the plant’s medicinal properties. Therefore, our study aimed to assess the local uses, management, and the production constraints of *Moringa oleifera* in Kilifi County, Kenya.

## 2. Results

### 2.1. Demographic Profile of the Respondents in Individual Interviews and Focus Group Discussions

Focus group discussion (FDG) is a research method used for collecting the opinions and perceptions of people regarding selected categories on a specific topic or issue. In this study, the participants were drawn from mixed age groups, with the middle-aged category (41–50 years) being the majority (40%) and young adults (21–30 years) being the minority (6.7%) (Table 1). Most of the participants in the FGDs were predominantly women (60%) compared to men (40%). In terms of tribal affiliation, the Giriama had the highest number of participants (40%), while the Chonyi had the lowest (16.7%).

The majority of participants (50%) derived their income from agriculture-related jobs, such as agricultural extension, farming, and field officer roles in non-governmental organizations. This was followed by those involved in business (30%), while the smallest group (20%) depended on other occupations.

In the individual interviews, the largest proportion of participants (32.9%) were seniors over 60 years old who were engaged in moringa farming (Table 1). Female-headed households accounted for more than two-thirds (71.1%) of the total households, while male-headed households made up the remaining third (28.9%). Among the ethnic groups, the Giriama had the highest number of respondents (64.5%), while the Rabai had the lowest (9.2%). Regarding occupation, the majority of respondents (30%) were involved in business-related jobs, including roles such as hoteliers, hotel employees, shopkeepers, taxi operators, tailors, and vendors. In contrast, other jobs alongside salaried individuals represented the smallest group, each accounting for 6.6%.

More than half of the respondents (61.8%) had divided their agricultural land into two distinct portions for kitchen and home gardening. While home gardens were much larger and principally used for growing trees, crops, and herbs, the kitchen gardens were smaller in size. More than half of the respondents (61.8%) lived closer to market centers and had small pieces of land, while the rest (38.2%) lived far from market centers and had relatively larger land sizes capable of accommodating kitchen gardens, home gardens, and crop fields. Therefore, the participants’ land size determined the land use in the area. On average, the respondents possessed 1.19 acres of land for crop, tree, or animal production.

### 2.2. Knowledge and Experience of Moringa Cultivation

All of the respondents interviewed confirmed that they were familiar with moringa and its production (Table 2). Less than a quarter of the respondents (18%) knew the plant as moringa, while a few of them (2%) referred to it as mzungwi. We found that most (82%) of the respondents knew the plant locally as mzungi; hence the name was more dominant in the region compared with the other local names. Our study did not find any specific meaning attached to these names. Most of the respondents (55.3%) had between 1 and 5 trees in their compounds, and only 5.2% had more than 20 trees, indicating that the plant was less preferred by residents compared to locally grown major crops. Regarding plant varieties, most respondents (96.1%) were familiar with only one variety, but a few (3.9%) recognized the existence of two distinct varieties based on taste and morphological traits, particularly leaf size. According to the respondents, the narrow-leaved plants were more bitter compared to the broad-leaved ones.

### 2.3. Management Practices and Production Constraints of Moringa

The majority (84.2%) of respondents used seeds to propagate the plant, compared to a smaller number (15.8%) who used seedlings (Table 3). Most respondents (81.6%, and 5.3% respectively) obtained seeds from their neighbors and relatives, while only 2.6% sourced seeds from their trees. Seedlings were mostly obtained from neighbors and relatives (3.9%, and 6.6% respectively), with the lowest number coming from their own selected trees (2.6%). All respondents noted that the plant required management practices, such as watering, especially during the early stages, and occasional pruning for proper growth. Slightly more than half of the respondents (51.3%) identified pests (birds, termites, and worms) as a significant challenge in growing the plant, particularly during dry spells. The majority of respondents (60.5%) were not comfortable with the unpleasant taste of the leaves, which hindered their consumption.

Slightly more than half of the respondents (53.9%) reported that improving the taste of the leaves was necessary, while the remainder (46.1%), mostly older individuals, believed that altering the taste could interfere with the plant’s medicinal properties and was therefore unnecessary. More than a third of respondents (36.8%) felt that the small size of the leaves was a hindrance to effective harvesting, especially when a large quantity of leaves was needed, and suggested that enlarging the leaves could be beneficial. In contrast, the majority (63.2%) were satisfied with the current leaf size.

Regarding the effect of moringa on other plants, most respondents (96.1%) reported that it did not have any detrimental effects on other crops, while a few (3.9%) noted that its shade caused etiolation in commonly grown crops such as maize and beans.

### 2.4. Uses of Moringa in Kenya

The main uses of moringa, as reported by respondents, included human nutrition, medicinal value, firewood, and ornamental purposes (Table 4). Among these, medicinal value was the most significant benefit, as nearly all parts of the plant were used to treat various ailments. Most respondents stated that they grew moringa for its medicinal properties. They mentioned using different parts of the plant to treat a wide range of common ailments, including high blood pressure, diabetes, ulcers, and stomach aches. The leaves were used both as food and medicine for treating these ailments. To reduce the bitter taste, respondents reported cooking the leaves with pigweed (locally known as “muchicha”), tomatoes, and meat. Additionally, the plant was used for fuel, particularly the cut branches after pruning or when the plant was dried out.

### 2.5. Focus Group Discussion

#### Uses of Moringa and Its Associated Constraints in Terms of Production and Consumption

The majority of the participants stated that the plant played a significant role in their livelihoods, serving as a source of food, ethnomedicine, fodder, firewood, and live fences. They mentioned that the leaves, flowers, and pods were reliable sources of food even during famines and droughts. A 65-year-old woman stated: “Moringa is a good food; it is the only food we have when the crops dry up due to lack of rain and high temperatures”. Some of the respondents opined that though the leaves were a vital part of the plant with immense nutritional benefits, their consumption was constrained by the unpleasant taste and thus only consumed them when they felt sick.

A 35-year-old man asserted: “I see people eating moringa, but I do not eat it because it has a bitter taste”. Most of the respondents stated that the plant had medicinal benefits and thus healed them of multiple ailments. They mentioned that almost all parts of the plant (leaves, flowers, seeds, roots, bark, and resins) were locally used to treat various ailments (Table 5). Most of the participants, especially the older ones, described moringa as a potent plant that can cure ailments that are sometimes untreatable in health facilities and provided examples of friends and relatives successfully cured by the plant (Table 5). A 70-year-old man stated: “I developed high blood pressure and diabetes sometime back, and when I started using moringa, the ailments disappeared, and I’m now healthy”.

It was evident that the same constraints (the bitterness of the leaves, pests, and small leaf sizes) mentioned during the individual interviews were mentioned during the FGDs. The majority of participants stated that the bitterness of the leaves impeded the plant’s use as food. An average number of the respondents opined that pests (birds, termites, and worms) were a threat to moringa production in the area, especially during dry periods. Others mentioned that the leaf size was small, making it time-consuming to harvest a large volume for sale or to prepare a meal for many people. Most of the participants asserted that moringa grew well with crops without causing any harm.

## 3. Discussion

### 3.1. Moringa Cultivation and Management

The broad use of various morphological parts of moringa, as indicated among the Mijikenda people, has also been reported in other African communities. The tribe used the plant for food, ethnomedicine, fencing, and firewood. This presents a broader use of the plant among the tribe, as moringa in Kenya is only known for remedying ailments and is occasionally used as food [50]. Their use of the leaves, pods, and flowers is similar to that reported in previous studies by [51] in South Africa and India [52]. The community primarily embraced the plant as food and medicine, compared to its other potential uses. The use of the plant as food and medicine aligns with previous studies by [53,54] in Kenya, which reported moringa as a food and remedy for most ailments.

The preference for the leaves and seeds as potential food and medicine has been reported in studies by [29] in Nigeria and [50] in Kenya. The use of the plant parts by the Mijikenda people to treat various ailments, including high blood pressure, diabetes, ulcers, wounds, body pains, and male impotence, has also been reported in similar studies [50,53]. Previous studies have demonstrated various management practices in moringa cultivation. We found that the seeds and seedlings from neighbors, friends, or relatives were used for the propagation of the plant. We found that these planting materials were also sourced from the “farmer’s preferred plant”, which they considered ideal for desirable traits, such as its “good” taste. Our findings align with a previous study by [55] in Swaziland, which reported that farmers primarily cultivate moringa from seeds and cuttings. A related study by [12] reported that farmers in Botswana propagated moringa with seeds, seedlings, and cuttings.

The respondents stated that the plant requires minimal management practices, unlike major crops grown in the area such as maize, beans, sorghum, and cowpeas, among others. They mentioned that the plant’s main management practices were pruning, pest control, and watering, especially during the early stages after germination. This corroborates earlier studies by [55,56], who reported the same management practices.

Our study revealed that the plant regenerates easily from seeds and sprouts from cuttings or stumps. During our field visit to the study area, we observed that the plant was growing by the roadsides in Kilifi town and Matsangoni market center, which was an indication that it was capable of regenerating naturally. According to the respondents, there were two types of moringa in the study area, the narrow-leaved and the broad-leaved. Some of the respondents associated the narrow-leaved type with a more bitter taste, while the broader-leaved type was associated with less bitterness.

### 3.2. Uses of Moringa

The broad use of various morphological parts of moringa in the current study demonstrated that the use of the plant among the Mijikenda community is similar to previously reported uses in other African communities. The community uses the plant for food, ethnomedicine, fencing, and firewood, which reveals a wider use of the plant as, in Kenya, it is only known for remedying ailments and is occasionally used as food [51]. The mode of using the leaves, pods, and flowers is similar to that reported in previous studies by [29] in Nigeria, [56] in South Africa and India [57]. The community uses the plant mainly as food and medicine, while the other uses of the plant remains low frequent, which aligns with previous studies by [50] in Kenya.

Similar to our study, the preference for the leaves and seeds of *Moringa oleifera* as both food and medicine has been reported in studies by [29] in Nigeria and [50] in Kenya. The use of various parts of the plant by the Mijikenda people to treat a range of ailments, including high blood pressure, diabetes, ulcers, wounds, body pains, and male impotence, has also been documented in similar studies by [50,58] in Kenya and Ethiopia, respectively. These studies emphasize the plant’s significance as a valuable source of non-timber forest products (NTFPs).

A similar study by [54] in Swaziland revealed the broader potential of moringa in treating various ailments, such as body pains, high blood pressure, flu, diabetes, asthma, arthritis, skin diseases, wounds, stomach cramps, a weakened immune system, and wounds. Similarly, in Nigeria, [59,60] confirmed the high medicinal value of moringa, using it to treat fever, ear infections, eye infections, diabetes, high blood pressure, the common cold, male impotence, and skin diseases. Apart from its nutritional and medicinal properties, people use the plant to establish live fences around their compounds and dry their prunings in the sun, which serve as a source of firewood [29].

### 3.3. Mode of Preparation

The shade drying of leaves and grinding them for use as food and medicine by the Mijikenda community aligns with others who reported similar uses, such as [61]. The chewing of moringa seeds or the grinding of them into powder to treat ailments, including hypertension, diabetes, and ulcers, corroborates previous studies by [30,58] which reported the plant’s potential to cure these ailments. The preparation of the plant for consumption involved boiling the leaves with flowers and pods, or individually, as also reported in the study by [50] in Kenya and Ethiopia. Additionally, the plant’s root, in ground form, was used to make moringa for the treatment of high blood pressure, diabetes, and ulcers, which was further supported by a similar study by [62] in Nigeria.

The community mixes moringa leaves with other vegetables and meat to reduce the bitter taste. This has also been reported in other African communities, such as Nigeria [29]. The bitterness seems to be a significant constraint on moringa consumption within the community, as it deterred them from consuming the plant regularly. A similar study by [63] in Mauritius showed that the taste of the leaves and pods was a major impediment to moringa consumption. Furthermore, a study by [18] revealed the bitter taste of the plant as one of the challenges affecting its acceptance as food in Botswana. Taste plays a significant role in satiation and food intake, which explains why the plant is not only unpopular but also has low production and consumption levels compared to locally grown crops in the area and other areas where it is cultivated [18,50].

The differences in the level of bitterness as reported by the community could be attributed to possible differences in glucosinolate levels among the plants, as previously reported by [64]. Several studies have addressed the issue of taste by mixing moringa leaves with other food products. For instance, the Hausa, Fulani, Sabe, and Ibariba ethnic groups in Nigeria cook and mix the leaves with groundnut cake (Kwulikwuli) and other spices [29]. In addition to the bitter taste associated with the plant, some respondents noted that the small size of the leaves posed a challenge, especially during harvesting when large quantities are needed for either home consumption or sale.

## 4. Materials and Methods

### 4.1. Study Area

**Figure 1:**
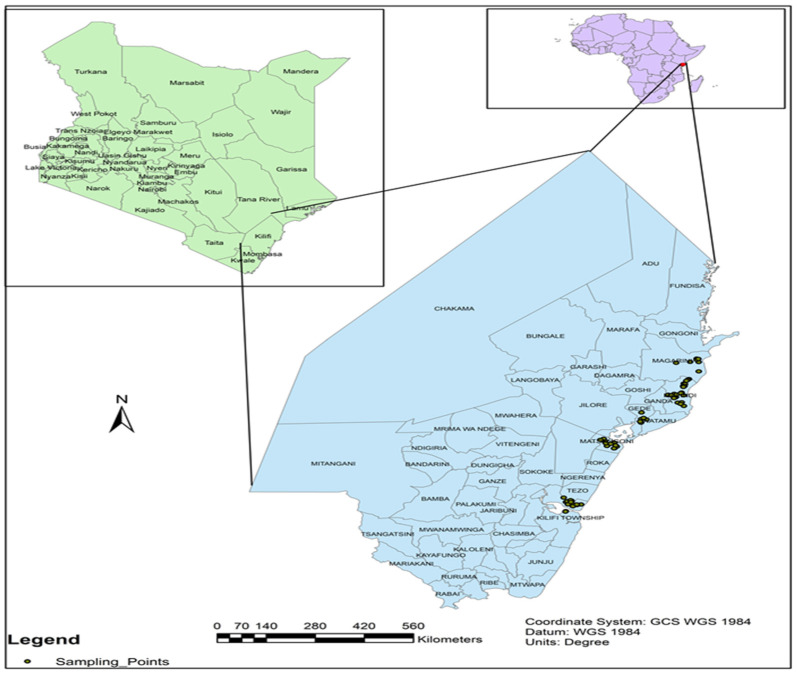
Map of Kilifi County showing the study sites.

We conducted an ethnobotanical field survey in Kilifi County, Kenya, between November and December 2022. The county is located in the coastal lowlands on a coordinate scale of 3.00230 S and 39.81670 E (Figure 1). It covers an area of 12,609.7 km^2^, has a population of about 1.45 million, and consists of 7 administrative sub-counties: Kilifi South, Kilifi North, Ganze, Malindi, Magarini, Kaloleni, and Rabai, with 35 devolved political units [61]. Rainfall is of a bimodal pattern, with average annual precipitation ranging from 300 mm to 1300 mm and average temperatures in the range of 21–30 °C [65,66]. Kilifi County borders the Indian Ocean to the east and rises to an altitude of 340 m above sea level to the undulating semi-arid plains that are suitable for ranching and tree crops. The rainy season occurs between March and May and from October to December, with peaks occurring in November and April. Clay, sandy clay, sandy loam, and clay soils are the dominant types [62].

### 4.2. Sampling

We sampled the study participants for both the FGDs and individual interviews in three sub-counties: Kilifi North, Malindi, and Magarini (Figure 1). In both the FGDs and individual interviews, we selected participants with extensive knowledge and experience in growing moringa based on a snowball sampling approach. The assistance of local leaders (assistant chiefs and county commissioners) was sought to identify moringa growers in the respective areas. We purposively selected three villages in each subcounty as follows: Kilifi North (Watamu, Tezo, Sokoni), Malindi (Furunzi, Shella, Soweto), and Magarini (Kagombani, Sambaki, Kibao).

### 4.3. Data Collection and Analysis

We conducted two focus group discussions (FGDs) involving a total of 30 participants. We held the first FGD in the Matsangoni ward, with 16 participants comprising 10 women and 6 men, and the second one in the Malindi town ward with 14 participants, consisting of 8 women and 6 men. The age of the participants in the two FGDs ranged between 21 and 60 years. All the participants claimed to use moringa regularly and were knowledgeable about its cultivation and management. The interviews were conducted in Swahili, which is the main spoken language in the study area.

**Figure 2:**
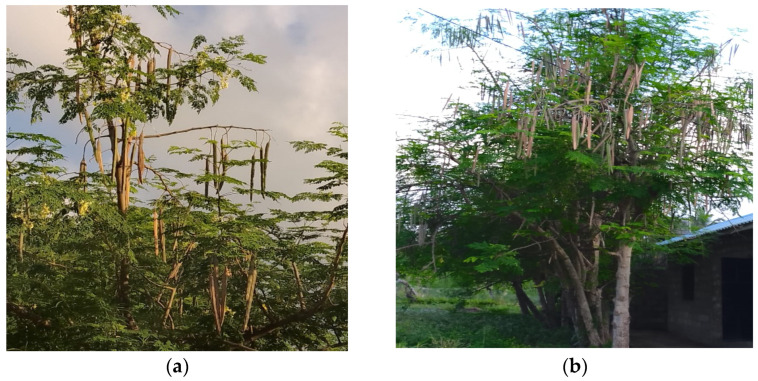
Moringa trees grown in Kilifi County near Malindi town: (**a**) mature tree with green leaves, dry pods, and flowers; (**b**) mature tree with green leaves and dry pods. Source: B.M.

Semi-structured interviews were employed with open-ended and closed-ended questions. The questionnaire had earlier been pretested, revised, and validated accordingly based on the field test. The study involved 76 people, comprising 22 men and 54 women, aged 21 to 80 years. These respondents were excluded from participating in the FDGs. We selected the respondents based on the presence of moringa trees in their homestead and their willingness to participate in the study (Figure 2). The purpose of the study was explained to the respondents before the interview. In exceptional cases, where respondents could not understand the language, a local person helped to translate the questions into the local dialect. Participants were asked to share information on the uses of the plant, the ways they currently utilize moringa, management practices applied, and possible constraints associated with the production and consumption of the plant. Data generated through the individual interviews were sorted and analyzed descriptively through percentages using the SPSS statistical package (ver. 20.0, SPSS, IBM Inc., Armonk, NY, USA).

## 5. Conclusions

The local people use the plant as a source of food, medicine, fuel, and a means of fencing their homesteads. All parts of the plant (leaves, flowers, bark, and roots) were locally used as food or medicine. The leaves were the most commonly utilized part of the plant, serving as food and medicine for treating common ailments, such as high blood pressure, diabetes, and ulcers. However, we identified several bottlenecks that hindered its production and utilization in the area, including the unpleasant taste of the leaves, which has made it less popular than locally grown exotic vegetables. The results indicated that the small size of the leaves poses a challenge in harvesting a large volume for either home consumption or commercial purposes. Pest infestations such as birds and bore worms hampered the plant’s production, particularly during hot weather. The study showed that most households had only one to five trees in their compounds, indicating a lower preference for the plant compared to major locally grown crops in the area. There is a need to create region-wide awareness of the potential benefits of the plant in the region. Furthermore, concerted efforts are needed to alleviate the plant’s production challenges.

## Figures and Tables

**Table 1:** Social demographic characteristics of the participants in FGDs and individual interviews.

Variable	Group	Individual Interviews		FGDs	
		No. of Respondents (*n* = 76)	Proportion of Respondents (%)	No. of Respondents (*n* = 30)	Proportion of Respondents (%)
Gender	Male	25	32.8	12	40.0
	Female	51	67.1	18	60.0
Age	21–30	4	5.3	2	6.7
	31–40	9	11.8	5	16.7
	41–50	18	23.7	12	40.0
	51–60	20	26.3	11	36.6
	>60	25	32.9	-	-
Ethnic group	Giriama	49	64.5	12	40.0
	Kauma	10	13.2	6	20.0
	Rabai	7	9.2	7	23.3
	Chonyi	10	13.1	5	16.7
Occupation	Farming	19	25.0	15	50.0
	Self-employed	23	30.2	9	30.0
	Salaried	17	22.4	-	-
	Others	17	22.4	6	20.0
No. of household members	1–4	33	43.4		
	5–9	41	54.0		
	≥10	2	2.6		
No. of years lived in the homestead	<20	45	59.2%		
	≥20	31	40.8%		
Type of agricultural plots/field owned	Home garden and kitchen garden	47	61.8%		
	Home garden, kitchen garden, and food crop field	29	38.2%		
Estimate of land size	<4000 m^2^	23	30.3		
	4000–8000 m^2^	47	61.8		
	>8000 m^2^	6	7.9		

**Table 2:** Knowledge and experience of moringa production in the study area.

Variable	Response	Proportion of Respondents (%)
Familiar with moringa	Yes	100.0
	No	0.0
Knowledge of growing moringa	Yes	100.0
	No	0.0
Awareness of other names for moringa	Yes	18.0
	No	82.0
Meanings associated with the names	Yes	0.0
	No	100.0
Local names of the plant	mzungwi	2.0
	mzungi	80.0
	moringa	18.0
Range of plants per household	1–5	55.3
	6–20	39.5
	21–38	5.2
Awareness of moringa varieties	No	96.1
	Yes	3.9
Varietal differences	Based on leaf size (a: small-leaved plants; b: relatively large-leaved plants)	3.9
	Small-leaved plants perceived more bitter than relatively large-leaved plants	3.9

**Table 3:** Moringa management practices and production constraints.

Variable	Response	Proportion of Respondents (%)
Source of moringa trees	Sowed seeds	84.2
	Planted seedlings	15.8
Source of seeds	Neighbors	81.6
	Relatives Own land (selected trees)	5.3 2.6
Source of seedlings	Neighbors Relatives	3.9 6.6
	Own land (selected trees)	2.6
Need for management	Yes	100.0
	No	0.0
Management practices applied	Pruning	100.0
	Watering (early stages of growth)	100.0
	Pest control	51.3
Challenges associated with growing moringa	Bitterness	60.5
	Small leaf size	36.8
	Pests	51.3
Need for improving the taste of leaves	Yes	53.9
	No	46.1
Need for increasing the size of the leaves	Yes	36.8
	No	63.2
Effects of moringa tree on crops	Yes	3.9
	No	96.1

**Table 4:** Use of *Moringa oleifera* in the study area.

Use Category	Parts Used	Mode of Preparation	Mode of Consumption	Purpose of Use
Food	Leaves	The leaves are boiled in water singly or mixed with onion, meat, tomatoes, or amaranth	Eaten with cuisines such as “ugali”	Human nutrition
	Flowers	Flowers are mixed with leaves and boiled or fried. They are also mixed with tomatoes, amaranths, or meat and fried together	Eaten with cuisines such as “ugali” and “chapatti”	Human nutrition
	Fruits	Immature pods are cut, and cooked	Eaten with cuisines such as “ugali” and “chapati”	Human nutrition
Medicine	Leaves	Leaves are dried and ground into a powder; Leaves are boiled in water (moringa tea)	Sprinkle the powder on prepared food such as “porridge tea” or meatsoup; Taken as a herbal tea	Treatment of high blood pressure, diabetes and, stomachache; Treats ulcers
	Seeds	Dry seeds are removed from pods and used immediately or dried further for long-term storage	Seeds are either chewed or ground into powder and sprinkled on food	Treats high blood pressure and diabetes
	Roots	Ground into powder and mixed with boiledwater and drunkas a tea	Taken as decoction	Relieves body pains, heal ulcers, treats high blood pressure and diabetes, relieves stomachache
Firewood	Trunks and branches	The fresh wood is sundried and used as firewood	Firewood for cooking	Firewood for cooking
Ornamental	Whole tree	Planting around the homestead	Live fences	Aesthetic

**Table 5:** Uses of the parts of the plant and constraints to consumption and production derived from FGDs.

Plant Part	Uses	Constraints to Consumption and Production
Leaves	Used as vegetables cooked and eaten with “ugali” (a traditional cuisine common in the study area). Eaten fresh or dried under shade and ground into powder to treat stomach disorders, backaches, headaches, body pains, diabetes, high blood pressure, ulcers, wounds, and male impotence	The small size of the leaf makes it difficult to harvest large quantities when needed as it requires a lot of time. Pests attack the leaves making their use difficult
Flowers	Dried under shade together with the leaves and the two are mixed and ground into powder for treating the abovementioned ailments	
Seeds	Seeds are chewed like groundnuts when dry for the treatment of diabetes and high blood pressure The seeds are also ground into powder and applied in soups, tea, and porridge	
Resins	It is applied externally to treat wounds	
Roots and bark	It is dried under the sun, ground into powder, mixed with water, and taken as tea to treat high blood pressure, stomach pains, diabetes, back pain, and ulcers	
Branches	The cut branches after pruning are dried under the sun and later used for firewood after drying	
Entire plant	Planted around the compound to make a live fence	

## Data Availability

Data will be available upon request.
